# High-Throughput Transcriptome Profiling in Drug and Biomarker Discovery

**DOI:** 10.3389/fgene.2020.00019

**Published:** 2020-02-05

**Authors:** Xiaonan Yang, Ling Kui, Min Tang, Dawei Li, Kunhua Wei, Wei Chen, Jianhua Miao, Yang Dong

**Affiliations:** ^1^ Guangxi Key Laboratory of Medicinal Resources Protection and Genetic Improvement, Guangxi Botanical Garden of Medicinal Plants, Nanning, China; ^2^ Dana-Farber Cancer Institute, Harvard Medical School, Brookline, MA, United States; ^3^ School of Life Sciences, Jiangsu University, Zhenjiang, China; ^4^ College of Biological Big Data, Yunnan Agricultural University, Kunming, China; ^5^ State Key Laboratory for Conservation and Utilization of Bio-Resources in Yunnan, Yunnan Agricultural University, Kunming, China; ^6^ School of Pharmacy, Guangxi Medical University, Nanning, China

**Keywords:** transcriptome, gene expression microarray, RNA-seq, natural drug discovery, biomarker discovery

## Abstract

The development of new drugs is multidisciplinary and systematic work. High-throughput techniques based on “-omics” have driven the discovery of biomarkers in diseases and therapeutic targets of drugs. A transcriptome is the complete set of all RNAs transcribed by certain tissues or cells at a specific stage of development or physiological condition. Transcriptome research can demonstrate gene functions and structures from the whole level and reveal the molecular mechanism of specific biological processes in diseases. Currently, gene expression microarray and high-throughput RNA-sequencing have been widely used in biological, medical, clinical, and drug research. The former has been applied in drug screening and biomarker detection of drugs due to its high throughput, fast detection speed, simple analysis, and relatively low price. With the further development of detection technology and the improvement of analytical methods, the detection flux of RNA-seq is much higher but the price is lower, hence it has powerful advantages in detecting biomarkers and drug discovery. Compared with the traditional RNA-seq, scRNA-seq has higher accuracy and efficiency, especially the single-cell level of gene expression pattern analysis can provide more information for drug and biomarker discovery. Therefore, (sc)RNA-seq has broader application prospects, especially in the field of drug discovery. In this overview, we will review the application of these technologies in drug, especially in natural drug and biomarker discovery and development. Emerging applications of scRNA-seq and the third generation RNA-sequencing tools are also discussed.

## Introduction

The research on lead compounds with novel structures, significant activities and clear mechanisms require multidisciplinary and systematic collaboration. The active ingredients of natural medicine have various skeleton structures and extensive biological activities (Winter et al., 2013; Mou et al., 2015; Lopez-Perez et al., 2017; Philkhana et al., 2017). Statistically, more than 1/3 of the new drugs on the market between 1981 and 2014 were directly or indirectly derived from natural products. The annual global medicine market in recent years is up to 1.1 trillion US dollars. About 35% of these medicines directly or indirectly originated from natural products: animals (~3%), microorganisms (13%), and plants (25%) (Calixto, 2019). A large proportion (7/26) of the antibacterial medicines approved from 2008 to 2018 is derived from natural products (Beutler, 2019). In synthetic drugs, there are also a considerable number of natural products borrowed from the skeleton structure or pharmacophore. In the area of cancer, from the 1940s to the end of 2014, 85 of the 175 small molecules approved by FDA were actually either natural products or derived from natural products (Newman and Cragg, 2016). Natural drug molecules such as morphine, penicillin, cephalosporin, aspirin, paclitaxel, and their derivatives have diverse structures and show various biological activities, making great contribution to the maintenance of human health (Brownlee and Woodbine, 1948; Clissold, 1986; de Weger et al., 2014). The above statistics indicate that the strategy of new drug development based on natural product sources is still dominant in the modern drug development process. The concept of “genomics” was first coined by Dr. Thomas H. Roderick, in 1986 (P.Yadav, 2007). Nowadays, the neologism “omics” informally refers to the field of biological research that ends in “omics,” such as genomics, transcriptomics, proteomics, or metabolomics, *etc*. The purpose of “omics” is to collectively characterize and quantify the pools of biomolecules that translate into structures, functions, and dynamics of one or more organisms. With the continuous advances in “-omics” technologies, the molecular mechanism of some diseases has been clarified and the scientific connotation of some active natural ingredients with unclear mechanism and target has been fully interpreted (Matthews et al., 2016; Sun and Hu, 2016). In particular, the rapid development of genomics provides more convenient conditions for the discovery and deeper study of natural drugs (Harvey et al., 2015).

**Figure 1:**
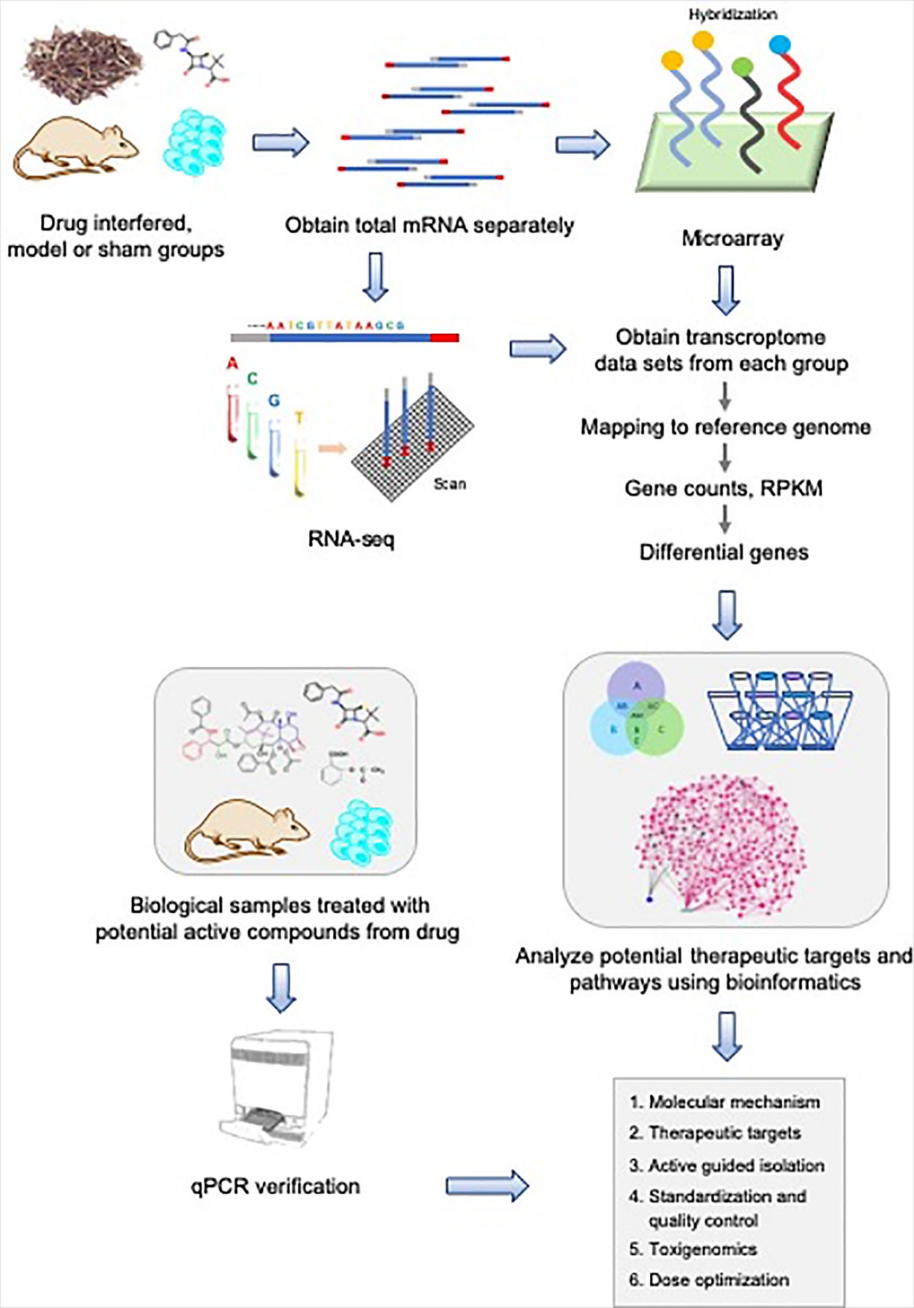
System-biology strategy for natural drug discovery based on transcriptome sequencing.

With the completion of the human genome project (Green et al., 2015), human beings have made unprecedented progresses in understanding and mastering own genetic information. At the same time, platforms with continuously improving transcript detection capability, especially in the higher molecular level, dramatically stimulate the rise of efficiency. In general, transcriptomics focus on the heterogeneity of the cell(s) transcriptome at the integral level in a specific time and space by high-throughput sequencing (HTS) technologies. HTS supporting transcriptomic research has experienced a series of developments including gene expression microarray technology (Schena et al., 1995), serial analysis technique for gene expression (SAGE) (Powell, 1998), massively parallel signature sequencing (MPSS) (Brenner et al., 2000), RNA sequencing (RNA-seq) (Wang et al., 2009; Behjati and Tarpey, 2013), single cell RNA sequencing (scRNA) (Xu et al., 2017), and several third generation sequencing approaches such as DropSeq (Macosko et al., 2015) and inDrop (Klein et al., 2015). These technologies reveal gene transcription level, regulation characteristics, and the molecular mechanism in the process of diseases and regulated pathways affected by drug intervention. These unique features facilitate the wide application of HTG in life and medicine sciences. Especially in the past 10 years, gene expression microarray and RNA-seq stand out for transcriptome profiling almost as a routine method (Chen et al., 2017). The employment of the two technologies on natural drug discovery is shown in **Figure 1**. The systematic character of the strategy makes transcriptomic sequencing can be applied in the following cases: firstly, to illuminate the molecular mechanism, composition or phytochemical components, and the potential therapeutic targets of a natural drug in pharmacodynamics. Secondly, to identify the genes related to drug sensitivity or resistance and predict the potential positive effects or side effect of a natural drug in pharmacogenomics. Here we mainly reviewed several regular transcriptomic sequencing technologies in recent decades with the focus on their application on natural drug and biomarker discovery (**Figure 1**).

## Gene Expression Microarray Technology

Gene expression microarray technology (also known as GeneChip, DNA/RNA chip, or BioChip), hereinafter referred to as microarray, is invented in the 1990s (Schena et al., 1995). This technique refers to the fixation of a large number of probe molecules (nucleic acids with known sequences) on the support and hybridization with labeled sample molecules. The number and sequence information of sample molecules are obtained by detecting the hybridization signal strength of each probe molecule (Gabig and Wêgrzyn, 2001). The basic steps of a microarray experiment include obtaining mRNA from appropriate biological samples, labeling the RNA or cDNA copies with fluorescence, hybridizing the labeled RNA or cDNA with microarray (up to millions of probes) for a while followed by washing off the excess, scanning the microarray under a laser, and analyzing data by appropriate software (Chavan et al., 2006). According to different detection purposes, gene chips can be categorized into SNP chips (John et al., 2004), copy number detection chips (Hehir-Kwa et al., 2007), RNA expression detection chips, and methylation detection chips (Kurdyukov and Bullock, 2016). The gene expression microarray technique platform mainly has the following characteristics:

1. The microarray-based gene expression platform can only be applied to quantify the gene expression with reference sequence.
2. Microarray detection can obtain large sample data information in a short time.
3. The accuracy of the differentially expressed genes screened by microarray is very high after verified by qPCR.
4. A single gene microarray chip can detect the whole genome of different types of RNAs, including mRNA, lncRNA, and circRNA.

## Application of Microarray in Natural Drug Discovery

### Microarray in Natural Drug Screening

The establishment of cell model provides convenience for studying the mechanisms of diseases and analyses of gene expression profiles throw helpful insights in the later studies. In addition, analyzing gene expression patterns in response to drug therapy can help determine which patients will response to specific therapy. The features of gene expression microarray allow it can be used in revealing the desirable and undesirable aspects of a drug, and the drug screening at cell or tissue levels can greatly reduce the usage of animals and the spending on experiments. Dooley *et al.* reported the application of DermArray^®^ and PharmArray^®^ DNA microarrays technology to detect gene expression in inflammatory bowel disease (IBD) tissue samples, and tested the effects of IBD drug treatments on gene expression in CaCo2 cells (Dooley et al., 2004). They verified seven genes from the over-expressed genes by RT-PCR (TMPT, FABP1, IFI27, LCN2, COL11A2, HXB, and metallothionein), which may become new candidate molecular target genes for IBD treatment and drug discovery. The effects of azathioprine, 5-aminosalicylic acid, metronidazole, and prednisone were found in another experiment. In azathioprine treated CaCo2 cells, the expression of metallothionein mRNA was found to be down-regulated, while in the Crohn's disease (CD) sample, the expression of metallothionein mRNA happened to be up-regulated, leading to an inverse correlation. These results of this study showed that the new method for drug screening is feasible.

### Microarray in Traditional Medicine Research

Crude extract, pre-fractionated extract, and pure compounds from medicinal plants or herbs are the three main sources for natural drug screening. These natural sources contain variety of molecules with potential bioactivities. However, it is difficult to elucidate the bioactivities of these natural extracts due to the complexity of the molecules and the possibility of interaction between the molecules. The high-throughput, large-scale and parallelism of gene expression microarray technology make it possible to be widely used in drug screening, especially in identifying the authenticity of traditional Chinese medicine (TCM) formulae, screening of effective ingredients, pharmacological mechanism research, and chemical drug synthesis (Gu and Chen, 2014; Ge et al., 2018a; Ge et al., 2018b). Moreover, the employment of microarray gene expression for large-scale screening in cell lines can shorten the screening time, determine the drug targets, and check the toxicity or side effects of drugs (Liu et al., 2015; Carrella et al., 2016; Hong et al., 2018; Rodrigues et al., 2019; Wang et al., 2019b).

TCM has been used for thousands of years to treat various diseases and developed numerous formulae. However, the formulae are difficult to be widely accepted by academia because the therapeutic mechanisms and the relationships between their ingredients are still not clarified. Cheng *et al.* reported the potential action mechanism of a formulae (San-Huang-Xie-Xin-Tang, SHXXT), and the relationship between the formulae and their ingredients in TCM by gene expression microarray and bioinformatics technology for the first time (Cheng et al., 2008). The TCM formulae of SHXXT consists of *Radix et Rhizoma rhei* (Dahuang), *Rhizoma coptis* (Huanglian), and *Radix scutellariae* (Huangqin), which has been used to treat gastritis, gastric bleeding, and peptic ulcers. They analyzed the mechanism of SHXXT and determined the relationship between SHXXT and its herbal composition in HepG2 cells by microarray technique. Gene set enrichment analysis showed that the anti-proliferation activity of SHXXT and its components in HepG2 cells through the p53 signaling, p53 activation, and DNA damage signaling pathway. Network analysis showed that p53 modulated most genes. In addition, hierarchical cluster analysis showed that the gene expression profiles of *Rhizoma coptis* and SHXXT were similar. These results could explain the underlying mechanism of SHXXT and why *Rhizoma coptis* is the main herb that plays a major role in SHXXT.

Besides cell lines, the expression microarray can also be used in drug research at the animal level. Yukmijihwuang-tang (YMJ, also known as luweidihuang-wang) is a memory or cognitive enhancer. YMJ consists of steamed *Rehmannia radix*, *Discoreae radix*, *Corni fructus*, *Hoelen*, *Mountain cortex radicis*, and *Alismatis radix*, and it has long been used in the treatment of diabetes and neurosis. Rho *et al.* reported that the YMJ derivatives (YMJd) could be formulated to enhance memory retention (RHO et al., 2005). They used cDNA microarray tools (Incyte, Inc., MO, U.S.A.) to identify the candidate genes responsible for enhancing memory and evaluate the specific gene expression patterns by real-time PCR in SD rat model of passive avoidance task. The mechanism of YMJd on enhancing the memory retention was estimated using the mRNA of rat hippocampus by cDNA microarray analysis. The expression levels of 27 genes were significantly changed. cDNA microarray assay demonstrated that the genes encoding transthyretin and pep-19 in YMJd treatment group were highly expressed. This result was also verified by real-time PCR. The investigation indicated that YMJd exhibited significant effects on memory enhancement and gene expression, which are related to the prevention of neuron degeneration and neuron growth events.

### Gene Expression Microarray in Elucidating the Pharmacological Mechanisms of Natural Products

A study characterized the possible mechanisms of anti-invasive effect of curcumin on highly invasive lung adenocarcinoma cells (CL1-5) by microarray analysis (Chen et al., 2004). The results exhibited that the invasive-related genes including matrix metalloproteinase 14 (MMP14), neuronal cell adhesion molecule, integrins 6 and 4 were inhibited while some genes encoding heat shock proteins (Hsp) such as Hsp27, Hsp70, and Hsp40-like protein were induced after treatment of curcumin below its sublethal concentration. These results were further confirmed at RNA and protein levels by real-time PCR, western blotting, and immunohistochemistry. The genetic control of tumor cell metastasis could be explored and the pathways for new anticancer drug development could be established by analyzing the anti-metastasis genes regulated by curcumin and comparing the invasive-related genes that have already been recognized.

Lu et al. revealed that the natural hydroxyapatite (NHA) extracted from pig bones exhibited osteoinductive effect on mouse bone mesenchymal stem cells (MSCs) by a complete process of gene expression microarray (Lu et al., 2014). Bioinformatic analysis and real-time PCR results showed that the expression trends of *Bmp2*, *Klf10*, *Spp1*, *Sox9*, *Twist2*, and *Omd,* which related to TGF-β, MAPK, Norch, and Wnt pathways were altered after NHA administration. This investigation revealed that the potential mechanisms of NHA in osteoinductive effect were modulation of cell growth, proliferation, and differentiation, these activated pathways interacted and coregulated MSCs growth and differentiation.

Terzioglu-Usak *et al.* studied the physiological responses of PC-3 cells treated with genistein at different concentrations based on gene expressed microarray. The results showed that at physiological concentrations (≤10 μM), genistein induced activation of CDKs, MAPKs, and RPSKs, leading to cell proliferation and decreased migration, while at higher concentrations (>10 μM) of genistein reduced TGF-β expression by negatively regulating SMAD 2/3, 4 which are the downstream TGF-β signaling cascade. This investigation revealed the potential mechanism of genistein at different concentrations, which has a guiding significance or clinical use of genistein (Terzioglu-Usak et al., 2019).

Recent studies have shown that hesperetin can increase the sensitivity of adriamycin in breast cancer cells. Hermawan *et al.* retrieved gene expression microarray data of hesperetin-treated NCI-60 cell from the COMPARE public library, and compared these data with the list of breast cancer resistance regulatory genes obtained from Pubmed. Further, KEGG pathway enrichment and molecular docking studies proved that hesperetin was a targeted inhibitor of ABL1, DNMT3B, and MLH1 (Hermawan et al., 2019). The results of this study provided the basis for the application of hesperetin in the clinical application of breast cancer chemical resistance.

Dong et al. studied the potential mechanisms of Sanguinarine (SAN) in BGC-823 cells by gene microarray and bioinformatic analysis. The results showed that SAN could inhibit the proliferation of BGC-823 cells by downregulating the expression of miR-96-5p and miR-29c-3p, and activating the MAPK/JNK signaling pathway (Dong et al., 2019).

Caffeic acid phenethyl ester (CAPE), the main polyphenol extracted from bee propolis has been found to inhibit the growth of a variety of tumors. Liang *et al*. analyzed differentially expressed genes in nasopharyngeal carcinoma (NPC) with or without CAPE treatment by cDNA microarray. Through bioinformatics analysis, CAPE was determined to specifically inhibit the NF-κB signaling pathway by inhibiting the transport of the p65 subunit from cytoplasm to nucleus, thus inhibited the proliferation and metastasis of NPC cells (Liang et al., 2019). This study reveals that CAPE may be used as a potential therapeutic compound for nasopharyngeal carcinoma therapy.

It easily can be seen from the above cases that gene expression microarray technology has made great progress in screening of active drugs, elucidating the composition of natural drugs, the identification of therapeutic targets and pathways, the optimization of formulae, and providing a theoretical basis for the utilizes of natural medicines.

## RNA-Seq Technology

As an important application of next-generation sequencing technology (NGS), RNA-seq has been developing rapidly in the last decade and has become an important approach for transcriptome analysis and quantitative analysis of gene expression in organisms (Gabig and Wêgrzyn, 2001; Cloonan et al., 2008; Mortazavi et al., 2008; Wang et al., 2008; Wang et al., 2009). The development of high-throughput sequencing technology marked by next-generation sequencing technology shows the following characteristics: the next-generation sequencing platform has increasingly larger detection throughput, shorter detection time, and lower detection cost. The third-generation sequencing platform has realized long fragment sequence detection, with a wider range of flux and detection. Using the RNA-seq to analyze the transcriptome sequencing of the organism can complement and expand the gene database of this species, obtain a large number of expressed sequence tags (ESTs) information, and discover some new functional genes, which is beneficial to the subsequent gene cloning and relevant molecular markers development. RNA-seq can also study the temporal and spatial expression of specific tissue or cell genes and explore some unknown small RNAs, which has been widely used in disease diagnosis, drug screening and pharmaceutical mechanism *etc*. RNA-seq technology has many advantages such as:

1. High resolution. RNA-seq can accurately distinguish individual bases, therefore problems such as background noise and cross-reaction caused by fluorescence analog signal can be effectively avoided.
2. High throughput. Through transcriptome sequencing technology, hundreds of millions of base sequences can be obtained, which can basically cover the whole transcriptome.
3. High sensitivity. The rare transcripts as low as a few copies in target cells can be detected by this RNA-seq technique.
4. Convenient to use. This technology can be used to analyze the whole transcriptome of various species and does not need reference genome or design specific probes before sequencing. Instead, RNA-seq can directly analyze the whole transcriptome.

## Application of RNA-Seq in Natural Drug Discovery

### RNA-seq in Discovering the Molecular Mechanism in Diseases

Discovering the molecular mechanism of disease is an important premise for the development of new target drugs. Transcriptome research can identify the structure and function of genes at the integral level. RNA-seq is a powerful tool for detecting differentially expressed alleles of transcripts in specific biological processes and can reveal new molecular mechanism of diseases as no reference genome is required. Nowadays, RNA-seq has been wildly used in the study of disease mechanism, clinical diagnosis, and drug development.

MYCN oncogene amplification is a marker of poor prognosis in patients with neuroblastoma disease. Schramm *et al.* identified key genes and pathways associated with MYCN by RNA-seq on the SOLiD V4 platform (Schramm et al., 2013). They studied MYCN-driven transcriptome in 20 cases of primary neuroblastomas and compared the results to those of *in vitro* inducible MYCN cell model (SH-EP MYCN-ER). The results showed that total 223 gene expression of MYCN-amplified tumors were distinct different from that of single-copy tumors. Pathway analysis suggested that MYCN could up-regulate the transcriptome level of mTOR related genes. In a validation test, the mTOR pathway was activated on the protein level in MYCN-driven neuroblastomas in mice and activation of MYCN in SH-EP MYCN-ER cells resulted in high sensitivity to mTOR inhibition was observed. In another instance, He *et al.* identified genes related to temporomandibular joint osteoarthritis (TMJ-OA) used RNA-seq technology by Illumina Hiseq 4000 (He et al., 2018). These genes included matrix-degrading enzyme (ADAMTS-8), complement C1qa, C3, and C5aR1, and some firstly reported genes in TMJ-OA such as circadian genes (*Per-2*, *Dbp*, *Npas2*, and *Arntl*). *Per-2* was upregulated by LIPUS at mRNA and protein levels. In a study on lung adenocarcinoma, four fusion genes (*WISP1*, *RAMP1*, *MYBL2*, and *GATA2*) of the human leukocyte antigen (HLA) family were identified by RNA-seq technology, indicating that the HLA family genes might be potential targets for the lung adenocarcinoma therapy. A deep RNA-seq of MCF-7 breast cancer cells demonstrated that Cyclin D1, EIF3A, and tumor protein d52-1 promoted proliferation or migration of McF-7 cells (Yamaga et al., 2013). Genetic mutations are the leading cause of autism spectrum disorder (ASD). Duan *et al.* integrated analyzed RNA-seq and microarray data sets in multiple ASD mouse model. They identified several key candidates that may play an important role in the pathogenesis of ASD, including SDC4, CP, S1PR1, UBC, PDYN, GRIN2A, GABRA2, and CAMK4. These results provide potentially important targets for understanding the molecular mechanisms of ASD (Duan et al., 2019).

These studies suggested that the RNA-seq allowed the identification of molecular mechanism in diverse diseases and can be used in target drugs development to treat the relevant diseases.

### RNA-seq in Identification of Drug-Target Genes and Drug Re-Purpose

Identification of potential drug target genes is an important step in drug discovery. Detection of drug-induced genome-wide gene expression changes can be achieved by RNA-seq. Saini *et al.* screened a series of biomarkers and candidate drugs associated to age-related macular degeneration (AMD). As a result, they found nicotinamide (NAM) can improve the disease-related phenotypes by inhibiting drusen protein, inflammation and complement factors, up-regulated nucleosome, ribosome, and chromatin-modifying genes through RNA-seq technology. Based on these findings, they suggested that NAM could be an effective drug in developing treatments for AMD (Saini et al., 2017). Rutin is a natural product widely found in fruits and vegetables and has significant anti-cancer effects. However, the anti-cancer signaling pathway of rutin has not been, so far, clearly elucidated. Nasrabadi *et al.* analyzed the transcriptome in human colon cancer SW480 cells by bioinformatics tools based on Illumina Hiseq 2000 platform, and constructed, filtered, and enriched the interaction between rutin modulated small RNA (miRNAs), long non-coding RNA (lncRNAs), mRNA, and transcription factor (TFs). The results showed that the cell metabolism activity was significantly reduced, and the cell cycle was inhibited below G1 phase in rutin-treated cells. Enrichment analysis of the miRNAs-lncRNAs-mRNAs-TFs network showed that these effects were mediated by changes in glucose, lipid, and protein metabolism, regulation of endoplasmic reticulum stress response, negative regulation of cell cycle processes, and induction of intracellular and extracellular apoptotic signaling pathways (Nasri Nasrabadi et al., 2019). Zhao *et al.* found that Celastrol alleviated cholestatic liver injury in mice through activation of sirtuin 1 (SIRT1), increasing the farnesoid X receptor (FXR) signaling pathway, and inhibiting the nuclear factor-kappa B (NF-κB) and P53 signaling pathways by RNA-seq technology (Zhao et al., 2019). Pan *et al.* compared the changes of gene expression in human non-small cell lung cancer (NSCLC) cell line H1299 cells after Polyphenon E (PolyE) treatment. RNA-seq results showed that activator protein 1 (AP-1) was highly expressed in cancer cells and inhibited by PolyE. RNA-seq analysis also showed that 32 down-regulated genes in H1299 cells contained direct AP-1 binding sites, indicating that PolyE triggered chemical prevention activity by regulating the AP-1 target gene (Pan et al., 2014).

Due to the complexity of some diseases, the pharmaceutical industry has paid more attention to omics-based polypharmacology in recent years according to their achievements on therapeutic effects through modulating disease molecular network *via* multi-targets on a systematic approach. Traditional herbal medicines are becoming increasingly important in treating some complex diseases. The Qishenkeli (QSKL) formulae consists of six herbs traditionally used in China for the routine treatment of cardiovascular disease (Wang et al., 2012). Through comparing the gene expression levels in the QSKL treated and the control animals, Li *et al.* found that 279 differentially expressed genes. Based on the transcriptome method, 80 landmark drugs, 155 potential pharmacological targets, and 57 indications were identified. The results proved that the combined approach is effective to explore the pharmacological targets of complex drug system.

In clinical research, much efforts have been made to drug re-purpose. In such projects, HTS usually were utilized to profile transcriptome characteristics. Genetically, adult acute myeloid leukemia (AML) can be classified to 20 subtypes based on different mutants and the 5-year overall survival rate is only 27% (Miller et al., 2016; Siegel et al., 2017). In order to screen the therapeutic targets in AML, Baccelli *et al.* interrogated 200 primary specimens with mubritinib, which is a well-known ERBB2 inhibitor originally developed to treat breast cancer. It turned out that chemotherapy-sensitive AMLs displaying transcriptomic hallmarks of hypoxia have resistance to mubritinib. They also revealed that mubritinib can cause the death of ERBB2^+^ cancer cells in AML subtypes by preventing oxidative phosphorylation (OXPHOS) as it is a direct and ubiquinone-dependent electron transport chain (ETC) complex I inhibitor. (Baccelli et al., 2019). HTS also can be used to help studying the various therapeutic effects of clinical drugs. Metformin is world-wide used agent for the treatment for type-2 diabetes mellitus and the patients show prominent therapeutic variability to this drug. Ustinova *et al.* showed the differential expression genes (DEGs) induced by metformin in healthy individuals. The DEGs were involved in the intestinal immune network for cytokine-cytokine receptor interaction and IgA production pathways (Ustinova et al., 2019). Extensive research has been conducted to explore the re-purpose of current clinic drugs. Namikawa *et al.* completed a study focusing on the development of antivirulence therapeutics to hvKP (hypervirulent *Klebsiella pneumoniae*). Rifampicin was proved to have strong anti-mucoviscous activity against hvKP by decreasing transcript levels of rmpA (regulator of mucoid phenotype A) and its regulated cps (capsular polysaccharide synthesis) genes, indicating its potential as a candidate antivirulence agent to cure hvKP (Namikawa et al., 2019).

### RNA-seq in Identification of Genes Involved in the Drug Resistance and Sensitivity

Chemotherapy is one of the most effective methods for cancer treatment at present, but chemotherapeutic drug-resistance often occurs, that leading to the failure of cancer treatment. A large proportion of cancer patients’ deaths are related to drug resistance (Mendonca et al., 2019). Since RNA-seq can help to determine how a disease develops, determine the reason why the drugs are affected, and identify new transcripts and splicing events (Li et al., 2019a; Li et al., 2019b; Schissler et al., 2019), it can be used to identify genes associated with drug resistance, as well as miRNAs involved in drug resistance regulation. Triple-negative breast cancer (TNBC) is one type of breast cancer that are extremely difficult to treat. Due to the lack of three kinds of receptors (estrogen receptor (ER), progesterone receptor (PR), and human epidermal growth factor receptor 2 (HER2), a large amount of molecular variability, as well as strong drug resistance, consistent results cannot be obtained for its treatment. Safa *et al.* determined and analyzed the differentially expressed genes and their biological function of two different TNBC drug-resistant cell lines (subtype B SUM159 and MDA-MB-231) after JQ1 and dexamethasone therapy by RNA-seq. They found that the cytokine-cytokine receptor interaction pathway in these two cell lines showed consistent significantly differentially expressed. It provided new ideas for the development of new drugs to treat TNBC (Shaheen et al., 2018).

The role of miRNAs in drug resistance has been demonstrated by a large amount of evidence (Kutanzi et al., 2011; Zhang and Wang, 2017). It is possible to identify miRNAs involved in the development of drug resistance by comparing the miRNA expression profiles of resistant and non-resistant cells through miRNA-seq and/or RNA-seq. Resistance to doxorubicin (DOX) is a common barrier to effective treatment of liver cancer. Zhang *et al.* investigated the role of miRNAs in DOX resistance of hepatocellular carcinoma (HCC) using Illumina sequencing platform (Zhang et al., 2013). They analyzed the expression profile of miRNAs in human HCC line (HepG2) and its drug resistant counterpart (HepG2/DOX). They found that the most miRNAs were down-regulated in HepG2/DOX cells. They further indicated the target genes for overexpressed and down-regulated miRNAs were RBM22 and UBE2Q1, respectively. Functional annotation suggested that these affected miRNAs mainly involved in MAPK signaling pathway. This study provided a general description of miRNA expression profile, which would help us to search for possible miRNAs to assist therapy to overcome DOX resistance, or to develop new drugs to avoid drug resistance.

### Single Cell RNA Sequencing for the Whole Transcriptome Analysis

As described above, HTS is very powerful and cost-efficient tool for genome-wide transcriptome analysis. However, it has several blemishes which are hard to overcome, for instance, little sample material ideally as small as single cells and sample tissues with multiple heterogeneous cells (e.g. circulating tumor cells) (Mortazavi et al., 2008). Moreover, the initial RNA-seq preferentially amplified the 3' ends of RNAs which causes cDNA amplification bias. In fact, the whole transcriptions in a single cell are good enough for conventional transcriptome analysis (Lindstrom and Andersson-Svahn, 2010). Thanks to the advancement in technologies for single cell isolation and amplification of transcriptome (Brehm-Stecher and Johnson, 2004; Allen et al., 2011; Streets et al., 2014; Salafi et al., 2016), the single cell RNA sequencing (scRNA-seq) technology to analyze whole transcriptome complexity in individual cells was first applied in 2009 (Tang et al., 2009). Currently, scRNA-seq is emerging as another high-throughput tool for transcriptome studies, contributing to a variety of fields in both medicine and basic research (Shapiro et al., 2013; Grun and van Oudenaarden, 2015; Kolodziejczyk et al., 2015). Nevertheless, developing suitable pipelines giving consideration to both experiments and computation is a big challenge (Kolodziejczyk et al., 2015; Stegle et al., 2015). In general, scRNA-seq undergoes five steps before sequencing: single cell capture, single cell lysis, reverse transcription, preamplification (cDNA), and library preparation. Some robust protocols for scRNA-seq were established as guidelines in recent years (Kolodziejczyk et al., 2015). In the phase of cell capture, fluorescence activated cell sorting (FACS) is the most economical and efficient approach to isolate numerous individual cells per minutes (Lindstrom and Andersson-Svahn, 2010). Its main advantage is that researchers can isolate customized individual cells from heterogeneous cell pool by labeling targeted cells with different fluorescent antibodies (Allen et al., 2011; von Boehmer et al., 2016). Incorporating FACS to scRNA-seq, Keren-Shaul et al. developed an integrated pipeline with a complete experimental and computational framework, namely MARS-seq2.0. It has multiple layers of quality assessment, error detection and correction, noise distribution and sequencing saturation, graphical presentation (Keren-Shaul et al., 2019). In order to increase the yield of full-length cDNA libraries, SmartSeq and SmartSeq2 developed by Pacelli *et al.* use improved reverse transcription and temple switching (Ramskold et al., 2012; Picelli et al., 2013). It turns out that they facilitate the detection of novel exon, alternative splicing, and allele-specific expression (ASE). Although more and more technological breakthroughs come up in scRNA-seq, there are still many challenges that have to be overcome, for instance, preamplification bias introduced by PCR used in SIRT, SC3-seq, SmartSeq, and SmartSeq2 or by *in vitro* transcription (IVT) used in CEL-seq (Hashimshony et al., 2012) and MARS-Seq2.0.

The most wide and successful application of scRNA-seq is on cancer researches (Navin and Hicks, 2011; Li et al., 2015; Zhang et al., 2016), especially in circulating tumor cells (CTCs), disseminated tumor cells (DTCs), and cancer stem cells (CSCs). It is very difficult to uncover their roles in tumor progression and metastasis as the very low frequencies in bone marrow or blood (Cristofanilli, 2006; Allard et al., 2014). An advantage is strong heterogeneity of CTCs is observed in cancer cell transition between mesenchymal and epithelial cell types (Yu et al., 2013; Jakabova et al., 2017). In addition, previous studies proved that the heterogeneity of biomarker expression are discordance between primary tumors and CTCs (Miyamoto et al., 2016). Recently, Cheng *et al.* presented a tool, namely Hydro-Seq, is for high-throughput CTCs analysis with a scalable hydro-dynamic scRNA-seq barcoding technique. They had identified drug targets for hormone and targeted therapies in breast cancer (Cheng et al., 2019). Another tool, scdNet, developed by Chiu *et al.* can be used to screen the differentially expressed key genes and their networks in scRNA-seq-based CTCs datasets (Chiu et al., 2018). In a word, transcriptome analysis with scRNA-seq provides more precise insights into monitoring processes underlying carcinogenesis and selecting target therapeutics.

In theory, scRNA-seq has more accuracy and efficiency comparing with the traditional RNAseq and analysis on gene expression patterns at single cell level has more potential to provide more information for drug and biomarker discovery (Valdes-Mora et al., 2018). In complex diseases, Gawel *et al.* proposed a single-cell-based strategy, namely MCDMs (multicellular disease models), to identify diagnostic and therapeutic targets. In their validated study based on scRNA-seq data from both of mouse and human models of arthritis mouse and models, they found that the network centrality of MCDM cell types are rich of gene variants associated with RA which can be prioritized as targets (Gawel et al., 2019). Moreover, scRNA-seq is more convenient and confident to monitor cancer progression. Jang *et al.* investigated molecular heterogeneity in multiple myeloma cells from 15 patients at different stages of disease progression. Forty-four overexpressed genes associated with poorer overall survival were screened, especially in MM patients treated with bortezomib. By this study, they also identified molecular pathways with the most significance during MM progression (Jang et al., 2019).

### Third Generation Sequencing Facilitates the scRNA-seq Development

With the innovation of sequencing instruments, the third generation sequencing technology promise to facilitate the transcriptome analysis on the transcription information integrity, accuracy, and high-resolution in the short future (Shapiro et al., 2013; Liu and Trapnell, 2016). To date, scRNA-seq cannot perfectly handle the technical PCR or IVT bias and yield absolute quantification as they are the basic biochemical method of scRNA-seq. Microfluidics platforms (Wu et al., 2014), including microdrop techniques (Klein et al., 2015; Macosko et al., 2015; Zilionis et al., 2017) and molecular barcoding techniques (Islam et al., 2014), is the best deputy of the third generation sequencing, which can best help scRNA-seq to overcome the obstacle. Two very recent approaches for single cell transcriptomics are the microwell-based Cytoseq method and droplet-based DropSeq method. The details of the protocols can be seen in reference (Fu et al., 2014a; Fu et al., 2014b). In a recent example, Marion *et al.* applied single cell technologies, Drop-based scRNAseq, to check cellular heterogeneity contributes to therapy resistance. The single cell analysis identified a pathogenic cellular module named GIMATS associated with resistance with anti-TNF therapy in ileal Crohn's disease lesions (Martin et al., 2019). Furthermore, engineers have been devoting their effort to combinational co-assay in scRNA-seq that joint two or more omics profiles with the hope to decrease experiment times and simultaneously survey more aspects of cellular biology. Cao *et al.* applied their in-house tool, sci-CAR, in thousands of single cells from human lung adenocarcinoma-derived A549 cells and adult mouse kidney. Then they jointed the profiles of chromatin and transcription to investigate the treatment of dexamethasone. As a result, their analyses demonstrated the advantage of single-cell co-assays over sole assay (Cao et al., 2018). Zhang lab developed a similar tool named SNARE-seq, which also got the same conclusion (Chen et al., 2019).

## Conclusions and Future Perspectives

As a practical matter, although there is a growing momentum of using sequencing platforms for transcriptome sequencing, microarrays are more popular in the case of large sample size, especially in clinical research and drug development, due to their high detection speed, simple and quick data processing, and relative low cost. Sequencing, by contrast, is dwarfed by the complex analytic methods and the diversity of data analysis strategies. Studies from the perspectives of prognosis prediction, safety evaluation, and transferability of gene label showed that the detection effects of microarray and sequencing is similar, with high consistency and repeatability. Therefore, the two platforms are technically comparable for precision medical testing from the current point of view, but the low-cost of gene expression microarray technology makes it more suitable for large-scale drug screening. In the detection of transcriptome expression, the 6G data volume of RNA-seq, which is mainly promoted in the market at present, is not as sensitive as the gene expression microarray in the detection of genes with low or medium expression abundance. However, RNA-seq has a significant advantage over gene expression microarray in the discovery of new target genes. The comparison of advantages between RNA-seq and gene expression microarray is shown in **Table 1**.

**Table 1:** The comparison between RNA-seq and microarray.

Technology	Microarray	RNA-seq
Detection methods	DNA hybridization	High-throughput sequencing
Species detection quantity	Limited	Extensive
Signal	Fluorescence analog signal	Digitized signal
Throughput	High	High
Novel genes detection	No	Yes
Alternative splicing sites detection	No	Yes
Detection repeatability	High	High
Resolution	Probe length	Single nucleotide

Original whole transcriptome amplification (WTA) had been applied in a cell to get the gene expression profile with microarrays before the advent of RNA-seq (Iscove et al., 2002; Kurimoto et al., 2006). Tang *et al.* reformed the single cell WTA using RNA-seq instead of microarray to identify transcripts not listed in the probe table (Tang et al., 2009; Tang et al., 2010; Wang and Navin, 2015). With the aid of matured and cost-decreasing single cell isolation methods, scRNA-seq, as an advanced version of RNA-seq, has greatly improved the high-throughput, sensitivity, and the detection ability of alternative splicing events (Zhao, 2019). Moreover, third generational sequencing (Ozsolak, 2012) techniques like microfluidics platforms is accelerating scRNA-seq to more applicable on many diverse fields including microbiology, neurobiology, development, tissue mosaicism, immunology, and cancer research (Navin, 2014). Its long-read sequencing feature offers is playing a huge part in medical genetics by characterizing the genetic variation and alternative splicing that are difficult to detect with the prevailing NGS techniques. Consequently, combining microfluidics platforms and scRNA-seq can promise to investigate genetic disorders with complete transcriptomic information (Mantere et al., 2019).

Indirect strategies of drug discovery depend on ultra-high-throughput screening. Although RNA-seq is a powerful technique to investigate drug effects using transcript abundance change as a proxy, the readings of current platforms for direct drug discovery are limited (Wang et al., 2019a). Recently, Ye *et al.* developed a drug discovery platform, digital RNA with pertUrbation of Genes (DRUG-seq), a massively parallel, automated, low-cost next-generation sequence-based approach, which can analyze the changes of the whole transcriptome under chemical and genetic perturbations, and it has been successfully applied in an industrial high-throughput screening (Ye et al., 2018).

In terms of future practical application, on one hand, microarray can continue to play its advantages of high detection flux, fast detection speed, and simple analysis. the further development of detection technology and the improvement of analytical methods, RNA-seq will have broader application prospects, especially in drug discovery. On the other hand, drug and biomarker discovery should rely on the integration of multiple omics studies, such as genomics, transcriptomics, epigenomics, and proteomics (Shapiro et al., 2013; Heath et al., 2016; Matthews et al., 2016).

## Author Contributions

All authors listed have made substantial, direct, and intellectual contribution to the work and approved it for publication.

## Funding

This work was support by the grants from Jiangsu University (19JDG039), Guangxi Science and Technology Research Project (GuiKeAA18242040, GuiKeAD17129044), and Key Project of TCM Modernization Research (2019YFC1711000).

## Disclaimer

The work described has not been published before; that it is not under consideration for publication anywhere else; that its publication has been approved by all co-authors, if any, as well as by the responsible authorities—tacitly or explicitly—at the institute where the work has been carried out. The publisher will not be held legally responsible should there be any claims for compensation.

## Conflict of Interest

The authors declare that the research was conducted in the absence of any commercial or financial relationships that could be construed as a potential conflict of interest.

The reviewer GW and handling Editor declared their shared affiliation.

## Abbreviations

HTS, high-throughput sequencing; TCM, traditional Chinese medicine; NGS, next generation sequencing.
